# Pamphlets

**Published:** 1875-07

**Authors:** 


A Clinical Contribution to the Treatment of Tubal Pregnancy. By T. Gaillard Thomas, M.D. New York; 1875. Pamphlet; pp. 11.

In this very remarkable case Prof. Thomas opened with the galvano-caustic knife the cyst situated to the left of the uterus, and extracted the fœtus, measuring six and a half inches in length. The placenta was but partially removed, when severe hemorrhage compelled the use of per sulphate of iron and the tampon. The patient made a good recovery.

---

On Spasmodic Urethral Stricture. By F. N. Otis, M.D., Clinical Professor of Genito-Urinary Diseases, College Physicians and Surgeons. New York; 1875. Pamphlet; pp. 15.

---

Relations of Ophthalmia to Practical Medicine. By William Thompson, M.D. Philadelphia: J. B. Lippincott & Co.; 1875. Pamphlet; pp. 27.

---

Cooper's Dictionary of Practical Surgery and Encyclopædia of Surgical Science: a review by Paul F. Eve, M.D. Nashville, Tenn. Pamphlet; pp. 12.

---

Injections of Tincture of Iodine into the Cavity of the Uterus in Hemorrhage after Delivery. By James M. Trask, M.D. New York: Wm. Wood & Co.; 1875. Pamphlet; pp. 15.

---

Annual Report of the Board of Health of the City of Pittsburgh for the year 1874. Pittsburgh, Penn.; 1875. Octavo; cloth; pp. 127.

---

Fourteenth Annual Report of the Cincinnati Hospital for 1874. Cincinnati, Ohio; 1875. Pamphlet; pp. 56.

